# Artificial Intelligence in Veterinary Imaging: An Overview

**DOI:** 10.3390/vetsci10050320

**Published:** 2023-04-28

**Authors:** Ana Inês Pereira, Pedro Franco-Gonçalo, Pedro Leite, Alexandrine Ribeiro, Maria Sofia Alves-Pimenta, Bruno Colaço, Cátia Loureiro, Lio Gonçalves, Vítor Filipe, Mário Ginja

**Affiliations:** 1 Department of Veterinary Science, University of Trás-os-Montes and Alto Douro (UTAD), 5000-801 Vila Real, Portugal; 2 Veterinary and Animal Research Centre (CECAV), University of Trás-os-Montes and Alto Douro (UTAD), 5000-801 Vila Real, Portugal; 3 Associate Laboratory for Animal and Veterinary Sciences (AL4AnimalS), 5000-801 Vila Real, Portugal; 4 Neadvance Machine Vision SA, 4705-002 Braga, Portugal; 5 Department of Animal Science, University of Trás-os-Montes and Alto Douro (UTAD), 5000-801 Vila Real, Portugal; 6 School of Science and Technology, University of Trás-os-Montes and Alto Douro (UTAD), 5000-801 Vila Real, Portugal; 7 Department of Engineering, University of Trás-os-Montes and Alto Douro (UTAD), 5000-801 Vila Real, Portugal; 8 Institute for Systems and Computer Engineering (INESC-TEC), Technology and Science, 4200-465 Porto, Portugal

**Keywords:** artificial intelligence, machine learning, deep learning, veterinary imaging

## Abstract

**Simple Summary:**

Artificial intelligence is emerging in the field of veterinary medical imaging. The development of this area in medicine has introduced new concepts and scientific terminologies that professionals must be able to have some understanding of, such as the following: machine learning, deep learning, convolutional neural networks, and transfer learning. This paper offers veterinary professionals an overview of artificial intelligence, machine learning, and deep learning focused on imaging diagnosis. A review is provided of the existing literature on artificial intelligence in veterinary imaging of small animals, together with a brief conclusion.

**Abstract:**

Artificial intelligence and machine learning have been increasingly used in the medical imaging field in the past few years. The evaluation of medical images is very subjective and complex, and therefore the application of artificial intelligence and deep learning methods to automatize the analysis process would be very beneficial. A lot of researchers have been applying these methods to image analysis diagnosis, developing software capable of assisting veterinary doctors or radiologists in their daily practice. This article details the main methodologies used to develop software applications on machine learning and how veterinarians with an interest in this field can benefit from such methodologies. The main goal of this study is to offer veterinary professionals a simple guide to enable them to understand the basics of artificial intelligence and machine learning and the concepts such as deep learning, convolutional neural networks, transfer learning, and the performance evaluation method. The language is adapted for medical technicians, and the work already published in this field is reviewed for application in the imaging diagnosis of different animal body systems: musculoskeletal, thoracic, nervous, and abdominal.

## 1. Introduction

Artificial intelligence is a branch of computer science dedicated to the creation of systems capable of performing tasks that generally require human intelligence. It is composed of a great number of subfields and techniques, one of them being machine learning [1]. Artificial neural networks have been used for decades, but only recent advances in the image-processing machine learning field, such as access to greater computer power and larger quantities of labeled images, have led to great progress in the study of medical image analysis [2,3,4]. Alex Krizhevsky et al. (2012) entered an annual international image classification competition using a deep convolutional network, and achieved a performance in image classification that had never been seen before when using traditional computer techniques [5]. Since then, multiple studies have been conducted in this area, leading to improvements in tasks such as image classification, object detection, and image segmentation [1].

The evaluation of medical images is very subjective and complex; therefore, the application of artificial intelligence and deep learning methods to automatize the analysis process would be of great value. Many developments have occurred in the field of human medical image analysis over the past few years; however, in the veterinary field, progress is also starting to happen [6,7].

This article aims to provide veterinarians with an interest in this field with definitions and information on machine learning and its components, including deep learning, convolutional neural networks, transfer learning, and performance evaluation methods in a simple way and using a language adapted to medical technicians, followed by a review of machine learning in the small animal imaging field.

## 2. Machine Learning

Machine learning (ML) is a field of artificial intelligence (AI) used for the analysis and prediction of data [1,6]. Instead of using explicit programming, the computer recognizes patterns in the given data in order to develop an algorithm with which to execute tasks [1].

There are two major types of machine learning: unsupervised and supervised learning [6,8]. In unsupervised learning, the data are not labeled; only the input data are presented to the computer, and the AI learns patterns in these data to determine the output. The computer uses techniques such as clustering to group similar patterns in images [9,10]. However, this model has some limitations, such as unpredictability since it does not receive previous patterns to guide it through the learning process, and good results are difficult to obtain [1]. In supervised learning, the computer is given labeled data (images with landmark annotations by expert humans in the field), known as “ground truth data”, to train the model [8,11]. The computer then learns a mathematical function that maps inputs to outputs based on the dataset pairs provided [12]. In the medical imaging field, the most common type of ML used is supervised learning [7].

This section introduces a classical type of machine learning model, artificial neural networks, as a way of illustrating the fundamentals of machine learning. The issue of overfitting is explored, highlighting its basis and some solutions. Following this, other models and techniques of machine learning aimed more at image analysis are presented, such as convolutional neural networks, transfer learning, object detection, and segmentation. Lastly, some ways of evaluating the model’s performance are described.

### 2.1. Artificial Neural Networks and Deep Learning

**Figure 1:**
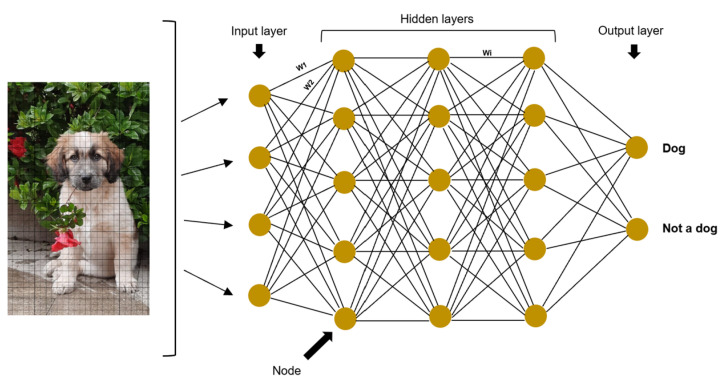
Architecture of an artificial neural network, in which the pixels of a digital image of a dog serve as input. There are four hidden layers and two possible outputs, “dog” or “not dog”. The nodes are arranged in layers and joined by connections. The weights are represented by the letter W (W1, W2, and Wi in the figure).

Artificial neural networks (ANNs) are a mathematical model used for machine learning, generally associated with supervised learning and inspired by the human nervous system [7,13]. They are formed by two principal components: the architecture and the weights [2]. This architecture is composed of nodes or neurons (the ANN’s basic units), which are arranged in vertical node layers. The layers are joined by connections so that each node has a connection with all the nodes in the following layer [8,14,15]. The first layer is the input layer, which receives the data to be analyzed, the last layer is the output layer, and between these two, there are hidden layers [9]. These layers are called hidden because neither the user nor the software has access to the results computed in them [7]. Each node in the hidden layers learns a different feature (i.e., curves, lines, brightness in a given image) [9]. The weights are numbers, usually randomly assigned and multiplied by each node, which are then adjusted in the process of network training [1,12]. Their purpose is to demonstrate the strength of one node’s influence on its neighboring nodes [7]. Then, the information passes through an activation function, and in the end, all the data are combined together to determine the final output [9]. For example, consider a network whose goal is to identify dogs in images: the input node would be the digital images; the hidden layers would be composed of nodes that consider different dog features such as typical lines or curves in the nose, eyes, ears, and fur; the weights would give different importance to each feature for the classification; and finally the output nodes would be “dog” or “not dog” (Figure 1).

Complex decisions require multilayer neural networks [14]. Neural networks with multiple hidden layers result in deep neural networks, that is, deep learning models [16]. The features learned by each layer are not determined by a human engineer, but determined by the model itself. The data scientist only needs to define the input layer, the number of hidden layers and nodes in each hidden layer, as well as the number of repetitions of the training [2]. Therefore, deep learning does not require much programming by humans and recognizing patterns in multi-dimensional data through examples [17,18]. However, increasing the number of hidden layers in deep learning means more computing power, and this makes it harder to understand the logic and interpretation of features used by the computer to achieve the output. This is called a black box problem [19]. Each node in the first hidden layer searches for something specific in the input layer but, entering deeper layers, the components become more and more abstract and complex than what a human would use to describe the same data [13].

The process of finding the weights that best fit the neural network is called optimization [9]. Forward propagation is the process of the AI reaching the predicted values, passing the input data through the model using an activation function, while backpropagation is the process of adjusting the parameters to minimize the loss function [15]. The loss function evaluates the difference between the ground truth and the model’s predicted values. The goal of the optimization is to minimize the loss function [15].

Gradient descent is one of the algorithms used to train neural networks to minimize the loss function [19,20,21,22]. Returning to the example of dog identification in images, this entails training the network to search for the best combination of weights (parameters) by presenting a set of images with and without dogs in order to obtain a final output of “dog” or “not dog”, respectively. This training has to be repeated a number of times and using the number of images necessary so that the identification error is minimized to a predefined acceptable error level.

### 2.2. Overfitting

Overfitting is a problem encountered in machine learning when a model fits too closely to a particular set of data and cannot be generalized to new data [10]. This happens when the model also fits the noise in the training data and lacks performance when presented with a new dataset [23]. To avoid overfitting, several measures can be considered when building a model: a large dataset, dropout, dividing the dataset, and stopping the train early (i.e., avoiding having too many iterations).

As large a dataset as possible should be collected to train the model, since reduced datasets can lead to overfitting [1,23]. Augmentation techniques can be performed to artificially increase data, transforming the dataset while keeping the same label. For this, the images can be blurred, rotated, zoomed, cropped, filtered, or the contrast can be modified, for example [15,24]. Active learning identifies the most useful data for the learning process to be annotated or labeled by an expert. It can therefore be helpful to maximize the model’s performance while using the least amount of data [25].

Dropout consists of removing a random percentage of the nodes on each training repetition [14]. This will prevent over-reliance on certain units and enable the system to be more robust even in the absence of certain nodes, allowing it to function in a more generalized way [21].

Dividing the dataset into training data (from which the model will learn) and testing data is also important in order to avoid overfitting and to assess whether the model can predict correct outputs when presented with different data [10]. The training data can be further divided into a training set and a validation set, where the validation set is a dataset used to evaluate and optimize the training [1]. The test data are used to evaluate the functioning of the model after the training sessions [10]. With this division, the model is trained to generalize, and not only to predict the data on which it was trained [15,23].

Stopping the training early is also important because while repeating it is necessary to reduce the error, too many iterations can lead to overfitting the model [9,26].

### 2.3. Convolutional Neural Networks

Convolutional neural networks (CNNs) are a type of deep learning model used mostly for image analysis [6]. They are generally composed of three types of layers: convolution, pooling, and fully connected [15,22].

**Figure 2:**
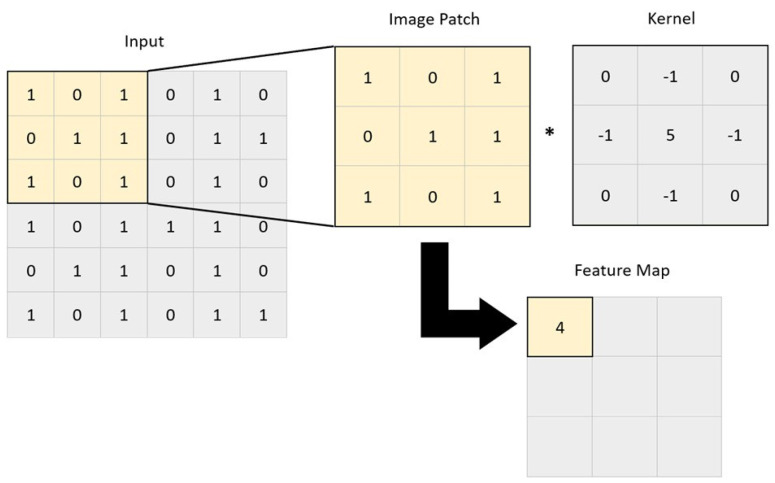
A convolutional operation, where a kernel is applied to a 3 × 3 set of neighboring pixels. A feature map is obtained by calculating the following expression: 1 × 0 + 0 × (-1) + 1 × 0 + 0 × (-1) + 1 × 5 + 1 × (-1) + 1 × 0 + 0 × (-1) + 1 × 0 = 4. *, represents the multiplication between the kernel and the image patch.

**Figure 3:**
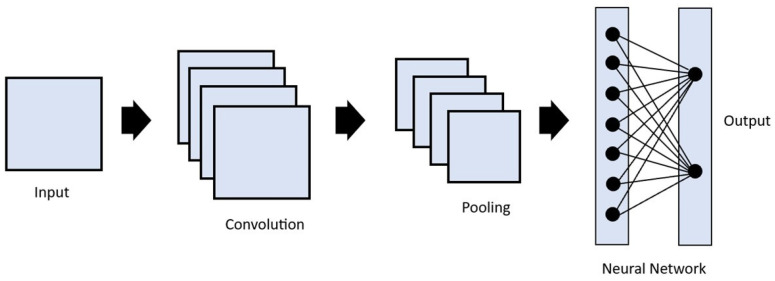
Representation of the architecture of a convolutional neural network. The input passes through a series of convolutional and pooling operations, then through a fully connected layer to determine the output.

Image convolution is a technique in which filters are applied to extract useful features in an image [9]. This is performed by applying a mathematical operation between a kernel matrix and an image patch (a section of the digital image). Each pixel in the patch region is multiplied by the values of the matrix, and then it is all summed up (Figure 2) [12]. This enables the image to be changed and emphasizes the relevant features, such as edges, different shapes, and blurred areas, enabling the model to transform the initial data into patterns that can be more easily identified by the AI [15,18]. The end result is a feature map resulting from the multiplications and additions, which passes through an activation function. The idea is that when a pixel is similar to neighboring pixels, they cancel each other out, leading to lower values appearing on the feature map, and if they are different, higher values appear in the feature map. The filters can be adjusted by altering the kernel matrix values based on the output error [12,18]. Entire image processing in a neural network is computationally expensive due to the great number of pixels used as input. The reduction in image size by sampling from regions in the input is thus a necessary step. This is called pooling or downsampling. The most commonly used form of pooling is max polling, where the pixel with the highest value is selected to represent a whole area [7,12,15]. There is also average pooling, in which the mean value of the pixels is used [7,15]. These two steps, convolution and pooling, are generally repeated multiple times, with each convolution layer being followed by a pooling layer. After this process, the resulting feature maps are flattened to reduce their dimensions and become a traditional neural network, which can have multiple hidden layers until the final output layer is reached (Figure 3). This final layer can function as a classifier, mapping the extracted features into outputs [12,15,21,22].

### 2.4. Transfer Learning

**Figure 4:**
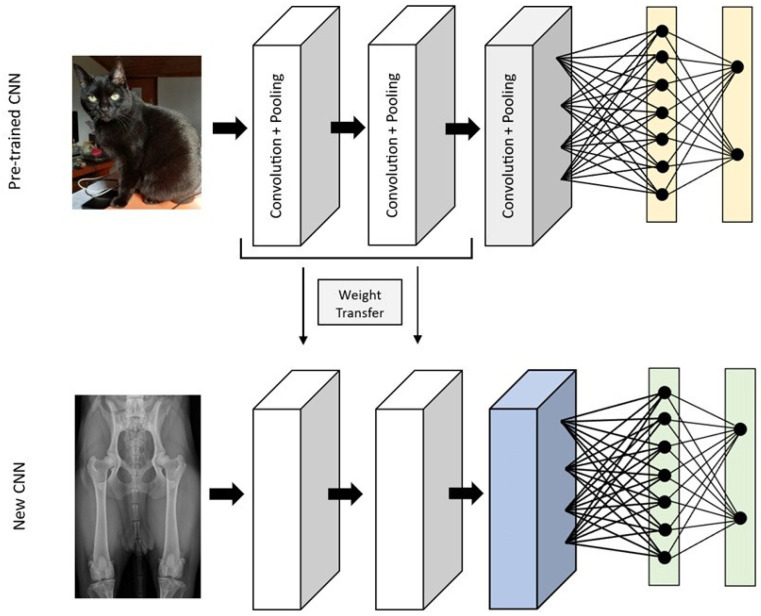
The transfer learning process, where part of the weights of a convolutional neural network trained to evaluate non-medical images, is used in a convolutional neural network to classify radiographs.

Transfer learning can be employed to overcome the problem of small datasets when using a CNN. Large datasets with thousands of images are necessary for effectively training deep learning models. However, obtaining such datasets in the field of medical imaging is exceedingly challenging [1,11]. This is both because there is a limited number of these images available to the public, and also because labeled data require annotations by experienced professionals, and therefore labeled data is even scarcer [3,17]. In transfer learning, a convolutional neural network model is pre-trained with other images in which the final layers are removed and replaced by the appropriate layers for the model [9,15]. Frequently, the model used is the ImageNet database if the network is fine-tuned with general images (such as images of animals, everyday objects, landscapes, and cars), but the network can also be pre-trained with medical images that were used for different classifications or tasks [9,15,27]. By using pre-trained networks, instead of starting the training with random weights, the weights of a similar model are transferred, which has been proven to obtain better performance and reduce the training time (Figure 4) [17,27]. The pre-trained model is already adjusted to detect features such as corners and shapes. Since these components are similar in all types of images, this already-created initial part of the model can be used and trained with the intended dataset, and the final part is adapted to our needs [15].

### 2.5. Object Detection and Segmentation Tasks

Object detection refers to the task of estimating the concept and localization of an object of interest within the images [28]. YOLO–You Only Look Once–is a recent open-source unified model for object detection. It localizes the region of interest in an image, that is, the region with the detail of an image that needs to be detected for a certain task. Previously described methods repurposed classifiers or localizers to perform object detection, applying the model to an image at multiple locations and scales. On the other hand, YOLO uses only a single convolutional neural network, processing the whole image at one forward propagation to obtain the classification and location simultaneously. YOLO divides the image into regions, predicting bounding boxes with an associated probability. By looking only once at the image, the network works much faster and is able to generalize better than other detection methods, because it understands the global context [29].

Image segmentation, also called pixel-based classification, is used to delimitate the boundaries of an area of interest [19]. The most commonly used deep learning architecture for this medical imaging segmentation is the U-Net. U-Net is a symmetrical, u-shaped network with a structure that consists of two parts: the first is called the encoder or contracting path, which functions as a standard CNN, with convolution and pooling layers to down-sample the images. The second part is called the decoder or expansive path, which uses deconvolutional operations to up-sample the images [19,30]. Between every encoder and decoder path, there are skip connections that link high-level features with low-level features, using a copy and crop operator, and resolving problems of special loss. This enables the network to increase the output’s resolution while learning localized classification [30].

### 2.6. Evaluation of the Model’s Performance

After training, it is crucial to evaluate the quality of the model built, to understand its performance [31]. To evaluate the model’s performance, metrics such as accuracy, precision, specificity, sensitivity, F1 score, and dice score can be used. These are obtained through parameters such as true negatives, true positives, false negatives, and false positives based on the prediction given by the AI model and the labeled data, which serve as the ground truth [32]. For classification tasks, there are also receiver operating characteristic curves (ROCs) and confusion matrices [15,31].

Accuracy is measured by the division between the correctly predicted data and the total number of predictions [31].

$$\mathrm{Accuracy}=\frac{\mathrm{True}\;\mathrm{positives}+\mathrm{True}\;\mathrm{negatives}}{\mathrm{True}\;\mathrm{positives}+\mathrm{True}\;\mathrm{negatives}+\mathrm{False}\;\mathrm{positives}+\mathrm{False}\;\mathrm{negatives}}$$

Precision is measured by the ratio between true positives and the sum of true positives and false positives. This indicates how accurate the model is in predicting positively [33].

$$\mathrm{Precision}=\frac{\mathrm{True}\;\mathrm{positives}}{\mathrm{True}\;\mathrm{positives}+\mathrm{False}\;\mathrm{positives}}$$

Specificity is the ratio between true negatives and the sum of the true negatives with false positives [32,34].

$$\mathrm{Specificity}=\frac{\mathrm{True}\;\mathrm{negatives}}{\mathrm{True}\;\mathrm{negatives}+\mathrm{False}\;\mathrm{positives}}$$

Sensitivity is one of the most important metrics for the medical field of machine learning. It is measured by the division between true positives and the sum of true positives and false negatives [33,34].

$$\mathrm{Sensitivity}=\frac{\mathrm{True}\;\mathrm{positives}}{\mathrm{True}\;\mathrm{positives}+\mathrm{False}\;\mathrm{negatives}}$$

F1 measure uses the precision and recall scores, combining the two into only one performance test [7,33].

$$F1=2\;\times \;\frac{\mathrm{Precision}\;\times \;\mathrm{Recall}}{\mathrm{Precision}+\;\mathrm{Recall}}$$

Dice score is generally used in segmentation. If the region of interest annotated by the expert and the one predicted by the model overlap completely, the score is one; if they do not overlap at all, the score is 0 [1,35].

**Figure 5:**
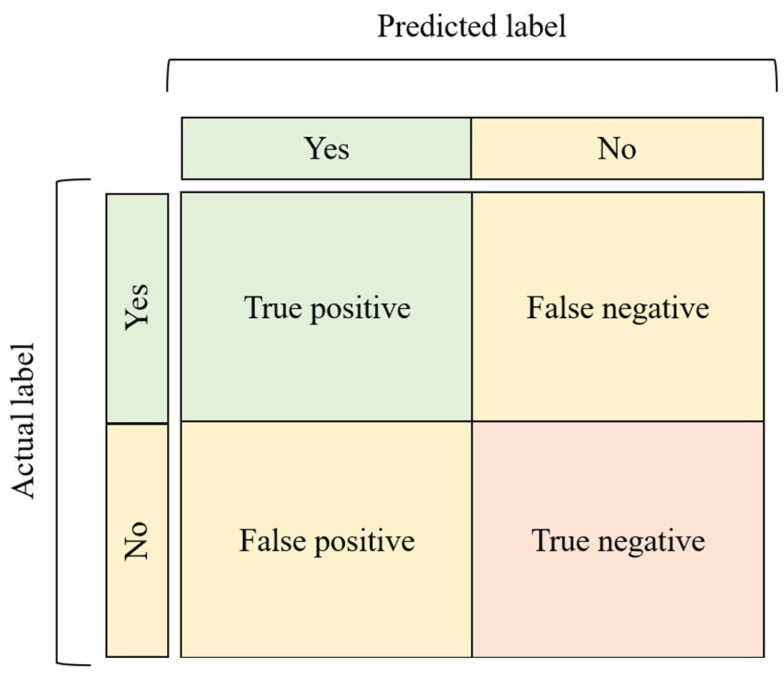
A confusion matrix, which facilitates the visualization of the model’s performance.

Confusion matrices are a way of visualizing the performance of the model by representing the counts from predicted and actual values in the form of a table (Figure 5) [1,33].

The ROC is a graphical representation of the model’s performance, with the true positive rate (or recall) on the *y*-axis and the false positive rate (or specificity) on the *x*-axis, which shows the performance for a number of different candidate threshold values between 0.0 and 1.0. With the ROC, the area under the curve (AUC) is calculated. This can vary between 0 and 1: when the value is 0.5, the model is unable to distinguish between two classes, and when it is 1, it predicts correctly 100% of the time [7,33].

## 3. Veterinary Imaging

Several authors have already applied machine learning technology in veterinary medicine. In this section, some of the advances made in small animal imaging using AI are presented (Table 1).

### 3.1. Musculoskeletal

McEvoy and Amigo (2013) were the first researchers to apply machine learning to the musculoskeletal region in the veterinary imaging field [7,36]. Firstly, in 2013, they used a partial least squares discriminant analysis model and an artificial neural network model to identify dogs’ hips in radiographs, classifying the images as “hip” or “not hip” [36]. Later, in 2021, McEvoy et al. used deep learning for the binary classification of hip dysplasia, in the first phase using a YOLO network to detect the hip region and then, in the second phase, to determine if hip dysplasia was present or not [11]. The obtained model was highly accurate [11]. Both studies showed that ML could be applied to veterinary imaging, specifically to hip dysplasia detection [11,36]. Gomes et al. (2021) carried out a similar study to McEvoy et al. (2021), using a CNN to classify dogs’ radiographs as dysplastic or not and measuring the model’s efficiency by comparing the results with the classification by an expert radiologist. Ultimately, the model and the veterinary radiologist produced similar results. Their work demonstrated that it is possible to use smaller datasets and still obtain accurate results by using transfer learning and pre-trained CNNs [39]. Akula et al. (2022) also applied CNNs for hip dysplasia, both in radiographs and in MRI, developing two models, one to identify canine hip dysplasia and another to classify the hips into FCI categories. The dysplasia detection model achieved good results, with an accuracy of 89.7%, whereas the classification model only achieved 70%. The small dataset could be one of the limitations of the study [31]. The Dys4vet group also used machine learning to create software to detect and classify hip dysplasia. Moreira da Silva et al. (2022) used a U-net for femur and acetabulum segmentation and active learning to maximize the model’s performance with the least amount of data. This led to the creation of a high-performing model which required 18.98% less annotated data [25,35].

Ergun and Guney (2021) used CNNs and compared the results with a support vector machine for the classification of radiographs to determine a dog’s maturity (accordingly to the growth plates), and also to detect fractures and date fractures in long bones and compare the results of each one. The group achieved good performance in all models, obtaining F1 scores from 0.62 to 0.89. This work also evaluated the effect of using data augmentation and transfer learning. Both were found to be useful, increasing the effectiveness of the models. However, the augmentation technique was shown to negatively affect the support vector machine model, although not the deep learning algorithms [40].

Ye et al. (2021) developed an automatic system to assist in the interpretation of spectral-domain optical coherence tomography of surgical margin tissue in dogs, using a CNN to classify the tissue as healthy or cancerous with high accuracy and precision [41].

Yang et al. (2015) and Duda et al. (2018) applied machine learning to magnetic resonance images (MRI) to identify muscular dystrophy in Golden Retrievers. Yang et al. (2015) used two different machine learning classifiers to classify the images as healthy or diseased. Duda et al. (2018) used three machine learning classifiers to classify the dystrophy progression in four phases. Both studies concluded that muscle texture analysis could be a promising tool. However, a larger dataset and other methods should be considered [37,38].

### 3.2. Thoracic

Yoon et al. (2018) and Burti et al. (2020) both used CNNs to evaluate radiographs from dogs. Burti et al. (2020) used it to evaluate the presence or absence of cardiomegaly, while Yoon et al. (2018) used it to assess cardiomegaly, the presence of pneumothorax, pleural effusion, pulmonary patterns, and mediastinal shifts. Yoon et al. (2018) also applied a bag-of-features machine learning model in the same study, which performed worse than the CNN [43,45]. Dumortier et al. (2022) used CNNs pre-trained with human chest X-ray images to identify pulmonary patterns in cats’ radiographs, training 200 different networks, each one with different randomly chosen training and validation sets, in order to improve the model’s performance [53]. Banzato et al. (2021) also used CNNs with transfer learning, using two different pre-trained models, ResNet-50 and DenseNet-121, to test which would be more efficient. The goal was for the model to classify dogs’ thoracic radiographs, labeling them as unremarkable, cardiomegaly, alveolar, bronchial, and interstitial patterns, presence of masses, pleural effusion, pneumothorax, and megaesophagus. ResNet-50 performed better, obtaining an area under the receiver–operator curve of above 0.8 in all parameters except for bronchial patterns and mass identification [34]. This group also developed a similar study to evaluate cats’ radiographs, testing a ResNet-50 and Inception V3 CNN. Both networks had similar performances, with high accuracy, except for mass detection [49].

Zhang et al. (2021) used deep learning to determine the vertebral heart score by measuring 16 key points in the vertebra and heart, which was then used to evaluate if there was cardiomegaly on dog X-rays, with an average performance of 90.9% [50]. More recently, Jeong and Sung (2022) proposed a new automated cardiac index for dogs to improve the classical vertebral heart score, using an algorithm that combined segmentation and measurements. The results showed that this new method could be used to diagnose cardiomegaly at an earlier stage and with a high degree of effectiveness [52].

Li et al. (2020) used CNNs for the detection of left atrial enlargement, comparing the results with veterinary radiologists’ evaluations. They trained two models, one that valued accuracy more highly and another that valued sensitivity more highly. The results revealed that the performance of the model with the emphasis on accuracy achieved an identical accuracy and sensitivity to the radiologists, with a concordance of 85.19% between the two [46].

Marschner et al. (2017) used ML in computed tomography of the pulmonary parenchyma to diagnose pulmonary thromboembolism in dogs. The model was able to distinguish between healthy and abnormal lung tissue. However, it was not able to efficiently distinguish dogs with this pathology from dogs with other lung pathologies [42].

Ott et al. (2021) applied the concept of deep CNNs to develop an AI capable of detecting pulmonary coccidioidomycosis, a zoonotic disease, in dog radiographs, achieving high-performance results [48].

Arsomngern et al. (2019) developed a radiograph diagnosis application (Pet-X) to detect lung abnormalities in cats and dogs using CNNs, by mapping the lesions and classifying them as alveolar, interstitial, and bronchial. The software performed better in lateral position X-rays, which can be explained by the fact that ventrodorsal images present more noisy features. As for the lesion classification, bronchial and interstitial detection models showed poorer performances than the alveolar model [44].

Boissady et al. (2020) used an AI program, PicoxAI, to screen thoracic X-rays for 15 types of primary thoracic lesions in cats and dogs. They used three different CNNs with three different pre-trained models: one without pre-training, another one pre-trained with images from ImageNet, and another pre-trained with ImageNet followed by training with a dataset of human thoracic X-rays. The network pre-trained only with unspecialized data (ImageNet) achieved the best results. The best model was then compared with classification by veterinarians, comparing the error rate in both [47]. In 2021, Boissady et al. also used PicoxAI’s CNN to calculate the vertebral heart score, comparing the results obtained with the annotations of veterinary specialists in order to evaluate the model’s performance. The final results showed a high agreement [51]. Hespel et al. (2022) also evaluated the performance of the PicoxAI program. They compared the error of using four different CNNs with the error rates of 13 veterinary radiologists in the analysis of thoracic radiographs, classifying the images with 15 possible labels. The results varied depending on the label [54].

Kim et al. (2022) studied another AI application—“vetology”—and compared its analysis with veterinary radiologist evaluations in the diagnosis of canine cardiogenic pulmonary edema. The accuracy, sensitivity, and specificity of the model were above 90%. However, despite the negative predictive value of 99%, the positive predictive value was only 56%, with several images being diagnosed differently from the veterinary expert’s evaluation [55]. Müller et al. (2022) carried out a similar study comparing evaluations in the diagnosis of pleural effusion, obtaining 88.7% accuracy, 90.2% sensitivity, and 81.8% specificity [56].

### 3.3. Nervous System

Banzato et al. conducted three studies in which they used AI to analyze the nervous system in MRIs. In 2017, they used machine learning texture analysis to predict the histological grade in dogs’ meningiomas [57]. In 2018, they carried out a new study on meningioma grading, this time using two different CNNs, one pre-trained, and one without pre-training. The de novo CNN proved to be more efficient [58]. Another study using a CNN and transfer learning was conducted by Banzato’s group in 2018 in order to differentiate between canine glioma and meningioma [59]. In all these studies, it was concluded that machine learning was an effective tool for assisting clinicians in MRI analysis [58,59].

Spiteri et al. (2019) applied machine learning and support vector machines to identify Cavalier King Charles dogs with Chiari-like malformation-associated pain and syringomyelia by detecting distinguishing features in MRI [60].

Biercher et al. (2021) developed a CNN to identify several thoracolumbar spinal cord pathologies in dog MRIs, such as intervertebral disc extrusion, intervertebral disc protrusion, fibrocartilaginous embolism, syringomyelia, and neoplasia. The model showed successful results in the detection and distinction of all pathologies except for syringomyelia and neoplasia. The authors concluded that more data should help in correcting this issue [61].

Wanamaker et al. (2021) used texture analysis machine learning to differentiate and identify glial tumor cells and non-infectious inflammatory meningoencephalitis in MRI and found the designed model to be efficient. Wanamaker also tried to grade subtypes within the two diseases, but without much success [62].

### 3.4. Abdominal

Banzato et al. (2018) used a deep neural network and transfer learning in ultrasound images to detect the presence of diffuse degenerative hepatic diseases in dogs. The results were compared to evaluate the model’s accuracy with serum biochemistry and cytology, using histopathology results as ground truth. They concluded that the model was more efficient at predicting disease than biochemistry and cytology [63].

Shaker et al. (2021) developed a machine learning model for CT analysis in detecting canine hepatic masses and predicting their malignancy by evaluating their heterogeneity [64].

## 4. Conclusions

Machine learning in veterinary imaging diagnosis has mostly been applied to the thoracic region, with various studies on the identification of pulmonary patterns and cardiomegaly detection. In addition, some commercially available software, such as “PicoxIA”, enable abdominal, thoracic, and hip image analyses, although studies have only been published validating the thoracic analysis. “Vetology” is another clinically certified software, which was created for the analysis of radiographs of the thorax, heart, and lungs in dogs. There are also a few studies on the musculoskeletal region, mainly the hip, for the detection of hip dysplasia. The nervous system and the abdominal region are the least studied regions for ML in veterinary medicine.

The most commonly used type of machine learning is supervised learning, with expert radiologists first labeling the images to train the AI. CNNs are the most commonly used model for image analysis since they are the best model for this purpose and have been improved in recent years.

The number of images used varies greatly since the necessary number of required images varies depending on the type of machine learning method applied and depending on whether or not augmentation techniques or transfer learning are employed.

## Figures and Tables

**Table 1:** Machine learning in veterinary imaging diagnosis.

Body Region	ML Type	Image Dataset	Objective	Diagnostic Imaging	Reference
Musculoskeletal	Partial least squares discriminant analysis and an ANN	256 images: 200 for training and 56 for testing	Identification of the hip region	X-ray	[36]
	Principal component analysis and support vector machine	1993 images: 936 from diseased dogs and 1057 from healthy dogs	Identification of Golden Retriever Muscular Dystrophy	MRI	[37]
	Support vector machine, Adaptive boosting, and Monte Carlo feature selection	38 images: 5 from diseased and 5 from healthy dogs	Classification of Golden Retriever Muscular Dystrophy	MRI	[38]
	YOLO network (YOLO v3 Tiny ConvNet)	1835 images: 1686 for training and 149 for testing to the identification of the hip region	Identification of the hip region and hip dysplasia classification as normal or dysplastic	X-ray	[11]
	CNN (Inception-V3)	225 images: 165 images for training and 60 for testing	Hip dysplasia classification as normal or dysplastic	X-ray	[39]
	CNN (AlexNet, GoogLeNet, and ResNet-50) and multi-class support vector machine	1000 images to evaluate the dog’s maturity, 410 images for fracture dating, and 2027 for fracture detection	Determination of the dog’s maturity, fractures’ dating, and fractures’ detection in long bones	X-ray	[40]
	CNN (ResNet-50)	200 images: 80 of tissue with cancerous margins and 80 of normal tissue for training; 20 of tissue with cancerous margins and 20 of normal tissue for validation	Classification of surgical tissue margins as healthy or cancerous	SDOCT	[41]
	3D CNNs	1400 images: 800 for training, 400 for validation, and 200 for testing	Hip dysplasia classification as normal or dysplastic	X-ray MRI	[31]
	U-Net and Feature Pyramid Network (FPN)	138 images normal and dysplastic: 70% for training, 15% for validation, and 15% for testing	Segmentation of the dog’s femur and acetabulum bones	X-ray	[35]
	U-Net	202 images: 70% for training, 15% for validation, and 15% for testing	Active learning in the segmentation of the dog’s femur and acetabulum bones	X-ray	[25]
Thorax	Principal component analysis, partial least square discriminant analysis, and support vectors machines	35 images: 29 images from diseased dogs and 6 images from healthy dogs	Diagnosis of pulmonary thromboembolism in dogs	CT	[42]
	Bag of features and a CNN	3142 images for cardiomegaly (1571 normal and 1571 abnormal); 2086 images for pulmonary patterns (1043 normal and 1043 abnormal); 892 images for mediastinal shift (446 normal and 446 abnormal); 940 images for pleural effusion (470 normal and 470 abnormal); and 78 images for pneumothorax (39 normal and 39 abnormal)	Identification of cardiomegaly, pneumothorax, pleural effusion, pulmonary patterns, and mediastinal shifts	X-ray	[43]
	CNN (DenseNet-121)	2862 images: 80% for training and 20% for validation	Pulmonary lesions identification	X-ray	[44]
	CNN (Inception V3, Inception-ResNet V2, VGG-19, and ResNet-101)	1468 images: 1153 images for training and 315 images for testing	Detection of cardiomegaly in thoracic radiographs	X-ray	[45]
	CNN (Visual Geometry Group 16 network)	792 images: 711 images for training and 81 for testing	Detection of left atrial enlargement	X-ray	[46]
	CNN (DenseNet-“PicoxIA”—a commercial program)	15780 images: 90% for training and 10% for validation	Identification of 15 types of primary thoracic lesions	X-ray	[47]
	CNN (Inception, MobileNet, ResNet, VGG, and a four-layer network)	1174 images (65% from healthy dogs and 35% from diseased dogs) and training and test sets sorted using ten-fold cross-validation	Detection of pulmonary coccidioidomycosis	X-ray	[48]
	CNN (ResNet-50 and DenseNet-121)	3839 images randomly divided into training, validation, and test sets in the ratio of 8:1:1	Classification of dog’s thoracic radiographs as unremarkable, cardiomegaly, alveolar, bronchial, and interstitial patterns, presence of masses, pleural effusion, pneumothorax, and megaesophagus	X-ray	[34]
	CNN (ResNet-50 and Inception V3)	1062 images randomly divided into training, validation, and test sets in the ratio of 8:1:1	Classification of cat’s thoracic radiographs	X-ray	[49]
	CNN (HRNet)	2643 images: 1875 for training, 399 for validation, and 369 for testing	Determination of the vertebral heart score to identify cardiomegaly	X-ray	[50]
	CNN (DenseNet-121-“PicoxIA”—a commercial program)	60 images: 30 canine and 30 feline	Calculation of the vertebral heart score to identify cardiomegaly	X-ray	[51]
	U-Net (Improved Attention U-Net)	1000 images: 800 for training, 100 for validation, and 100 for testing	New automated cardiac index to improve the vertebral heart score and identify cardiomegaly	X-ray	[52]
	CNN (ResNet-50 v2)	500 images: 455 for training and validation and 45 for testing	Pulmonary patterns identification in cats	X-ray	[53]
	CNN (DenseNet-121-“PicoxIA”—a commercial program)	55780 images: 90% for training and 10% for testing	Classification of thoracic radiographs with 15 possible labels	X-ray	[54]
	CNN (“Vetology”—a commercial program)	481 images	Accuracy determination of the “Vetology” for cardiogenic pulmonary edema diagnosis	X-ray	[55]
	CNN (“Vetology”—a commercial program with VGG-16 CNN architecture)	4000 images: 2000 of pleural effusion and 2000 of normal patients for training	Accuracy determination of the “Vetology” for pleural effusion diagnosis	X-ray	[56]
Nervous system	Linear discriminant analysis	58 sets of MRI scans of dogs with meningioma, 27 for training and 31 for testing	Prediction of the histological grade in dog’s meningiomas	MRI	[57]
	CNN (AlexNet and scrDNN)	56 images: 60% for training, 10% for validation, and 30% for testing	Prediction of the histological grade in dog’s meningiomas	MRI	[58]
	CNN (GoogleNet)	80 images: 70% for training, 15% for validation, and 15% for testing	Distinction between canine glioma and meningioma	MRI	[59]
	Sequential floating forward selection, support vector machine	32 images: 10 normal, 11 with the malformation and clinical signs, and 11 with clinical signs without malformation	Identification of Cavalier King Charles dogs with Chiari-like malformation-associated pain and syringomyelia	MRI	[60]
	CNN	500 images: 375 images for training and 125 images for testing	Identification of thoracolumbar spinal cord pathologies in dogs	MRI	[61]
	Random Forest classifier	119 images: 80 images for training and 39 images for testing	Differentiation and identification of glial tumor cells and non-infectious inflammatory meningoencephalitis	MRI	[62]
Abdomen	CNN (AlexNet)	48 images: 70% for training, 15% for validation, and 15% for testing	Detection of diffuse degenerative hepatic diseases	US	[63]
	Quadratic discriminant analysis	40 images	Detecting canine hepatic masses and predicting malignancy	CT	[64]

ANN, artificial neural network; CNN, convolutional neural networks; CT, computed tomography; ML, machine learning; MRI, magnetic resonance imaging; SDOCT, spectral domain optical coherence tomography; US, ultrasound.

## Data Availability

Not applicable.
